# Long-Term Survival in IDH-Wild-Type Glioblastoma: Clinical and Molecular Insights From Two Exceptional Cases

**DOI:** 10.7759/cureus.110449

**Published:** 2026-06-08

**Authors:** Ezgi Gurlek Tumer, Sumeyye Tuysuz, Efe Yetisgin, Yildiz Guney

**Affiliations:** 1 Radiation Oncology, Ankara Etlik City Hospital, Ankara, TUR; 2 Pathology, Ankara Etlik City Hospital, Ankara, TUR

**Keywords:** glioblastoma, idh–wild-type glioblastoma, long-term survival, molecular profile, radiotherapy

## Abstract

Glioblastoma is the most common and aggressive primary malignant brain tumor in adults, associated with poor prognosis despite standard multimodal treatment. Long-term survival beyond five years is rare, particularly in IDH-wild-type disease. We report the cases of two female patients with IDH-wild-type, CNS WHO grade 4 glioblastoma who achieved survival exceeding five years following standard therapy. The first case was a 51-year-old woman presenting with seizures and a left temporoparietal glioblastoma. She underwent gross total resection followed by radiotherapy (60 Gy) with concurrent and 12 cycles of adjuvant temozolomide (200 mg/m²). Due to high PD-L1 expression, nivolumab was added during adjuvant treatment. Molecular analysis revealed PTEN, TP53, and TERT promoter mutations. She remains disease-free at five years. The second case involved a 27-year-old woman presenting with headache and a left frontal glioblastoma. She underwent gross total resection followed by standard chemoradiotherapy and 12 cycles of adjuvant temozolomide (200 mg/m²). Next-generation sequencing (NGS) identified multiple alterations in DNA damage response and tumor suppressor pathways. She also remains in complete remission at five years. These cases highlight that long-term survival in IDH-wild-type glioblastoma is possible, although rare. Favorable clinical factors such as young age, good performance status, and gross total resection, together with distinct molecular profiles, may contribute to prolonged survival. These observations underscore the importance of integrating clinical and molecular features to refine prognostic assessment in glioblastoma.

## Introduction

Glioblastoma is the most common primary malignant brain tumor in adults, accounting for approximately 80% of all malignant gliomas [1]. The median age at diagnosis is around 65 years, and the disease shows a slight male predominance, with a male-to-female ratio of approximately 1.5:1 [2]. Despite advances in multimodal therapy, glioblastoma remains associated with a dismal prognosis. Radiotherapy constitutes a central pillar of glioblastoma management, providing a survival advantage and improved local disease control in patients treated with maximal safe surgical resection followed by concurrent and adjuvant temozolomide. Nevertheless, despite these benefits and completion of standard-of-care treatment, the reported median overall survival remains approximately 14 months. Long-term survival is rare; fewer than 5% of patients survive beyond five years [1]. Characterizing these exceptional long-term survivors may provide important insights into tumor biology, treatment responsiveness, and potential prognostic factors that could inform management strategies for the broader glioblastoma population.

The 2021 World Health Organization (WHO) classification of central nervous system tumors introduced major changes in the classification of diffuse gliomas. In the 2016 WHO classification, glioblastomas with IDH mutations, often referred to as secondary glioblastomas, were associated with a better prognosis and longer survival compared with IDH-wild-type tumors. Median overall survival was approximately 27.5 months in IDH-mutant cases and around 14 months in IDH-wild-type cases [3-4]. Therefore, the presence of an IDH mutation was considered a positive prognostic factor in glioblastoma.

In the 2021 WHO classification, IDH-mutant glioblastomas were reclassified as “astrocytoma, IDH-mutant, CNS WHO grade 4.” According to the current criteria, a diagnosis of glioblastoma requires confirmation of IDH-wild-type status. In IDH-wild-type diffuse gliomas, the presence of microvascular proliferation or necrosis is sufficient for the diagnosis of glioblastoma. In addition, even in the absence of these histopathological findings, the presence of at least one of the following molecular alterations, TERT promoter mutation, EGFR amplification, or combined chromosome 7 gain and chromosome 10 loss (+7/−10), is also sufficient for a diagnosis of ‘glioblastoma, IDH-wild type, CNS WHO grade 4 [5].

In this report, we present the cases of two female patients diagnosed with IDH-wild-type glioblastoma, as defined by the WHO 2021 molecular classification, who achieved overall survival exceeding five years following standard-of-care treatment incorporating radiotherapy. By situating these cases within the existing literature, we aim to contribute to the growing body of evidence challenging the uniformly fatal paradigm of glioblastoma and to highlight the need for integrative, biology-driven prognostic models by exploring potential clinical and molecular determinants of prolonged survival in this aggressive disease.

## Case presentation

Case 1

A 51-year-old woman presented in 2021 with new-onset seizures. Brain magnetic resonance imaging (MRI) demonstrated a 5-cm contrast-enhancing mass in the left temporoparietal subcortical region without midline crossing. Her Karnofsky Performance status (KPS) at diagnosis was 90, and no significant focal neurological deficit was present. The patient underwent gross total resection, which was confirmed by an early postoperative MRI obtained within 48 hours. Histopathological examination established the diagnosis of glioblastoma, IDH-wild-type, CNS WHO grade 4 (Figure 1).

**Figure 1:**
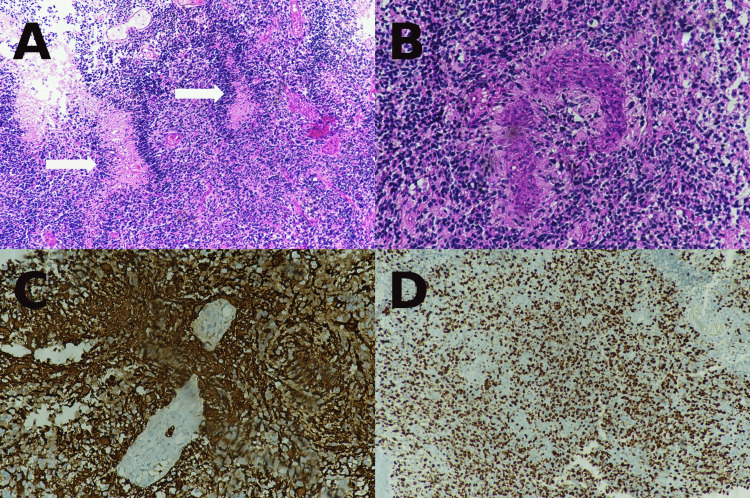
Histopathological and immunohistochemical features of the glioblastoma. (A) Hematoxylin and eosin–stained section shows a highly cellular glial neoplasm with marked nuclear atypia and pseudopalisading necrosis (white arrows) (×100). (B) Higher magnification demonstrates microvascular (endothelial) proliferation with multilayered endothelial cells (×200). (C) Immunohistochemical staining for glial fibrillary acidic protein (GFAP) reveals diffuse cytoplasmic positivity in tumor cells, confirming glial differentiation, while areas of microvascular proliferation remain unstained (×200). (D) Ki-67 immunostaining shows a high proliferative index, estimated at approximately 25% (×100).

Molecular profiling with next-generation sequencing (NGS) revealed alterations in PTEN, TP53, and a TERT promoter C228T mutation. Immunohistochemistry demonstrated high PD-L1 expression, with staining observed in more than 80% of tumor cells. MGMT promoter methylation was not detected. The Ki-67 proliferation index was approximately 25%.

Adjuvant external beam radiotherapy was initiated on postoperative day 20 and delivered using an intensity-modulated radiation therapy (IMRT) technique to a total dose of 60 Gy in 30 fractions to the tumor bed. Clinical target volume (CTV) was delineated based on T2-weighted fluid-attenuated inversion recovery (T2-FLAIR) abnormalities with an additional 1-cm margin, yielding a CTV of 183 cc. A planning target volume (PTV) margin of 3 mm was applied. Treatment was administered concurrently with temozolomide at 75 mg/m² (Figure 2). Corticosteroid therapy was not required during radiotherapy. Seizure control was achieved with levetiracetam.

**Figure 2:**
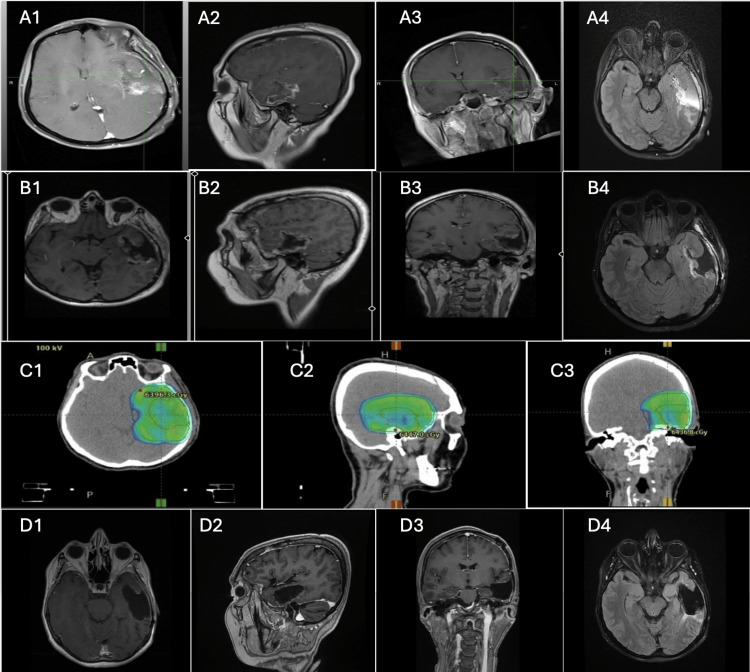
Preoperative, postoperative, last follow up MRI and radiotherapy treatment planning A1–A4: Preoperative MRI of the patient in different planes; (A1–A3) T1-weighted post-contrast images, (A4) T2-FLAIR image. B1–B4: Postoperative MRI of the patient in different planes; (B1–B3) T1-weighted post-contrast images, (B4) T2-FLAIR image. C1–C3: Radiotherapy treatment planning images; the 95% isodose line is shown in green, indicating adequate target volume coverage. D1-D4: Last follow-up MRI of the patient in different planes; (D1–D3) T1-weighted post-contrast images, (D4) T2-FLAIR image. T2-FLAIR: T2-weighted-fluid-attenuated inversion recovery

Following completion of chemoradiotherapy, the patient received adjuvant temozolomide (200 mg/m²) for 12 cycles. Given the high PD-L1 expression, nivolumab was administered concomitantly, due to physician preference, with adjuvant temozolomide for a total of 12 cycles. The treatment course was well tolerated without significant toxicity.

The patient has remained free of disease recurrence for five years since diagnosis. Serial follow-up MRI has demonstrated stable findings without evidence of progression. At the most recent evaluation, she remains in clinical and radiological remission with a KPS of 90.

Case 2

A 27-year-old female patient presented in 2021 with a headache localized to the left side of the head accompanied by nausea. Brain MRI revealed a 5.5 cm cystic lesion in the left frontal lobe with a 4 cm solid component demonstrating diffuse contrast enhancement. The lesion was solitary and without midline crossing. Her KPS status at diagnosis was 90.

The patient underwent gross total resection, which was confirmed by early postoperative MRI. Histopathological evaluation established the diagnosis of glioblastoma, IDH-wild-type (NGS confirmed), CNS WHO grade 4 (Figure 3).

**Figure 3:**
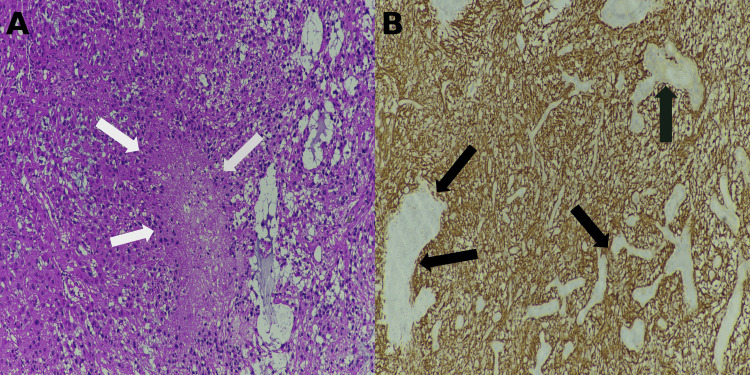
Histopathological and immunohistochemical findings (A) Glioblastoma showing pseudopalisading necrosis (white arrows) (H&E, ×100). (B) Glial fibrillary acidic protein immunostaining demonstrating diffuse tumor cell positivity with interspersed non-staining areas corresponding to microvascular proliferation (black arrows) (×100).

NGS identified multiple genomic alterations, including mutations in NF1, TP53, PIK3R1, ATM, BRCA1, FANCA, POLE, SMARCB1, ATRX, HRAS, SMARCA4, DDX3X, ERCC2, PBRM1, RAD50 and VHL.

Adjuvant external beam radiotherapy was initiated on postoperative day 15 and delivered using an IMRT technique to a total dose of 60 Gy in 30 fractions to the tumor bed. CTV was delineated based on T2-FLAIR abnormalities with an additional 1 cm margin, yielding a CTV of 160 cc. A PTV margin of 3 mm was applied. Treatment was administered concurrently with temozolomide at 75 mg/m² (Figure 4). Following completion of chemoradiotherapy, the patient received adjuvant temozolomide (200 mg/m²) for 12 cycles. No additional systemic therapy was administered.

**Figure 4:**
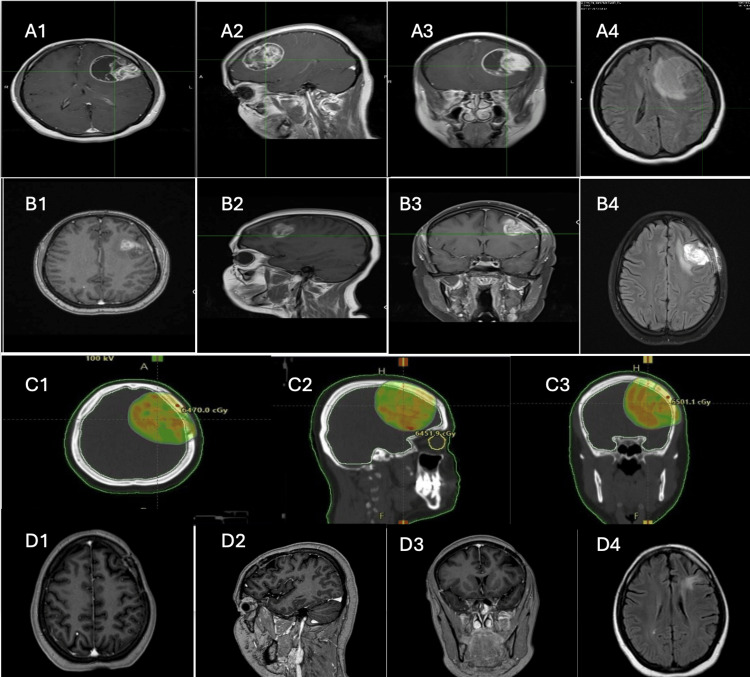
Preoperative, postoperative, last follow up MRI, and radiotherapy treatment planning A1–A4: Preoperative MRI of the patient in different planes, (A1–A3) T1-weighted post-contrast images; (A4) T2-FLAIR image. B1–B4: Postoperative MRI of the patient in different planes, (B1–B3) T1-weighted post-contrast images; (B4) T2-FLAIR image. C1–C3: Radiotherapy treatment planning images; the 95% isodose line is shown in green, indicating adequate target volume coverage. D1-D4: Last follow-up MRI of the patient in different planes, (D1–D3) T1-weighted post-contrast images; (D4) T2-FLAIR image. T2-FLAIR: T2-weighted-fluid-attenuated inversion recovery

The patient has remained free of disease recurrence for five years since diagnosis. Serial follow-up MRI has demonstrated stable findings without evidence of progression. At the most recent follow-up, she remains in clinical and radiological remission with a KPS of 90.

## Discussion

Despite the implementation of the Stupp regimen, long-term survival rates in glioblastoma remain limited, with 10-year survival reported at approximately 1% and five-year survival around 5% [6]. Several strategies have been investigated to improve outcomes beyond the standard of care. Dose-dense temozolomide was evaluated in the Radiation Therapy Oncology Group (RTOG) 0525 trial, while the addition of bevacizumab in the adjuvant setting was investigated in the RTOG 0825 and AVAglio trials; however, none of these approaches demonstrated a significant improvement in overall survival [7, 8].

Similarly, the phase III CheckMate 548 trial showed no survival benefit with the addition of nivolumab to radiotherapy plus temozolomide, and the SPECTRO-GLIO trial demonstrated no overall survival advantage with magnetic resonance spectroscopic imaging-guided dose-escalated radiotherapy [9, 10]. These findings underscore the limited progress achieved with multiple large-scale clinical trials in glioblastoma.

Tumor-treating fields (TTF), when administered in combination with the Stupp regimen and continued for up to two years or until disease progression, have shown an overall survival benefit of approximately five months. However, this benefit is dependent on high compliance, requiring usage for more than 18 hours per day, which may limit its practicality in routine clinical use [11]. 

Following primary treatment, recurrences are frequently observed, with approximately 80% occurring within 2 cm of the original tumor site [12]. Management options in the recurrent setting include re-resection, bevacizumab, CCNU, re-irradiation, and TTFs. In the RTOG 1205 trial, the combination of bevacizumab and re-irradiation demonstrated an improvement in three-month progression-free survival; however, it did not result in a significant overall survival benefit [13].

Survival beyond five years is uncommon and occurs in a small subgroup of patients with IDH-wild-type glioblastoma. Therefore, understanding the clinical, molecular, and genetic features of these long-term survivors is particularly important.

In patients with IDH-wild-type glioblastoma, prognosis is determined by a complex interplay of clinical and molecular factors. Established clinical predictors include the extent of surgical resection, particularly achievement of gross total resection, KPS, neurological functional status, and age at diagnosis, all of which are incorporated into widely used prognostic classification systems. On a molecular level, alterations such as EGFR amplification, TERT promoter mutation, chromosome 7 gain, chromosome 10 loss, PTEN loss, and CDKN2A/B homozygous deletion are generally associated with more aggressive tumor biology and poorer outcomes [5]. In contrast, MGMT promoter methylation remains the most robust favorable predictive biomarker, correlating with enhanced responsiveness to temozolomide and prolonged survival [14].

In the first case, the patient was older than 50 years, which is recognized as an adverse prognostic factor in glioblastoma. Nevertheless, the patient had a good performance status, which is associated with more favorable outcomes. Gross total resection was achieved, representing another positive prognostic factor. Considering these variables, the patient would be classified as class IV according to the RTOG recursive partitioning analysis (RPA) for glioblastoma [15], a subgroup in which the five-year survival rate has been reported to be approximately 4%.

Pembrolizumab monotherapy has demonstrated limited efficacy in recurrent glioblastoma, with low response rates and no consistent correlation with PD-L1 expression [16]. In our case, the tumor proportion score (TPS) for PD-L1 expression was 80%. However, in contrast to previously reported findings, the patient achieved durable disease control. The combined positive score (CPS) was not available for this patient. These findings suggest that, in selected patients with high PD-L1 expression, immune checkpoint inhibition may be associated with meaningful clinical benefit. The prolonged disease control observed in this case may represent one of several possible contributing factors, including but not limited to nivolumab therapy, rather than indicating a definitive causal relationship.

In the second case, the patient was younger than 50 years and had a good performance status; therefore, according to the RTOG RPA classification for glioblastoma, the patient would be categorized as class III, a subgroup in which the five-year overall survival rate has been reported to be approximately 14%. Furthermore, multiple genomic alterations were identified in this patient. High tumor mutational burden has been associated with improved outcomes in certain contexts [17]. The relatively favorable overall survival observed in this patient may, at least in part, be related to this molecular profile.

The clinical course of our second patient, who demonstrated a cystic component and relatively prolonged survival, is consistent with previously reported findings in the literature [18]. In a recent systematic review and meta-analysis, cystic glioblastomas were shown to have a significantly better overall survival compared to non-cystic glioblastomas, suggesting that the presence of a cystic component may be associated with a more favorable prognosis.

Previous studies investigating long-term survivors of glioblastoma have reported TP53 as the most frequently mutated gene in this patient population [19]. Consistent with these findings, both of our cases harbored TP53 mutations. This observation is in line with the possibility that TP53 alterations may contribute to the molecular features associated with prolonged survival in GBM, although further studies are needed to clarify this relationship.

## Conclusions

In summary, these two cases highlight that long-term survival in glioblastoma is likely the result of a multifactorial interplay between clinical characteristics, such as age, performance status, and extent of resection, and molecular features, including TP53 mutations and tumor mutational burden. However, given the limited number of cases, no definitive conclusions can be drawn regarding the prognostic impact of these factors or the contribution of specific therapies. The marked heterogeneity observed among long-term survivors underscores the need for further comprehensive, multi-institutional studies incorporating genomic, epigenetic, and immunological profiling to better elucidate the determinants of exceptional survival and to guide personalized therapeutic strategies in this challenging disease.
